# Observations on the Supposed Relations between Epizootics and Epidemics, and the Effects Which Diseased Meat May Have on Human Health

**Published:** 1858-05

**Authors:** George Sutton

**Affiliations:** Aurora, Dearborn County, Indiana


ART. II.—*Observations on the Supposed Relations between Epizootics and Epidemics, and Experimental Researches to ascertain the Nature of the recent Epizootic among the Swine, and the Effects which Diseased Meat may have on Human Health.* By GEORGE SUTTON, M.D., of Aurora, Dearborn County, Indiana.

IT is scarcely necessary to offer an apology for presenting to the medical profession a few facts in relation to the remarkable epizootic which has recently so extensively prevailed over different sections of our country, when we bear in mind that in all ages the opinion has been entertained that such wide-spread diseases among the lower animals arise from the same causes which spread pestilence and death among the human race; and that it is possible that the investigation of epizootics may throw some light upon the nature of the epidemic influence.

The long periods which sometimes occur between remarkable epizootics; the general opinion that such diseases are produced from the same causes which give rise to epidemics; the obscurity that still shrouds the causes which spread epidemics and epizootics over the globe; the uncertainty whether the epidemic influence extends over all animal life or is confined only to species; the little that is known of the nature of these widely extended diseases among inferior animals; whether they resemble any of the diseases to which the human system is subject, are certainly sufficient reasons to give to the investigation of epizootics an interest worthy of more attention from the medical profession than they have generally received. Epizootics and their etiology are as much parts of the great system of nature as the organization and physiology of animals; and while the energies of the most brilliant minds have been directed to unfolding comparative anatomy, epizootics and the different branches of comparative pathology appear to have received but a superficial attention; still it cannot be doubted that the investigation of these subjects may throw light upon human maladies, as well as enlarge our knowledge of the operations of those mysterious influences which spread diseases among the animal kingdom.

The accounts of epizootics which have come under my notice are extremely brief and unsatisfactory. We are informed by historians, that at different periods diseases have prevailed among inferior animals to such an extent as sometimes to even threaten the extinction of species. But what the nature of these diseases were, how nearly they resembled the epidemics which in many instances were prevailing at the time, and whether these accounts were not frequently exaggerated, (as we know there is a tendency in the human mind to magnify events which occur during pestilential periods,) are subjects of conjecture, and give to the investigation of epizootics at the present time additional interest. That periods remarkable for fatal diseases among inferior animals have, however, occasionally occurred, we have an abundance of testimony; and it is possible, although we have no record of such an event occurring within the historic period, that such wide-spread diseases may have brought about the extinction of some of the many thousands of extinct species of animals that we find beneath our soil or imbedded within our rocks. A brief review of some of the principal epizootics will not only show that they have been noticed in all ages, frequently during the prevalence of epidemics, and may probably afford some interest at the present time, when one of the most remarkable which has appeared within the last century has operated so extensively over the country.

We read in the Bible of the pestilence that was to cut off man and beast, and the murrain that was sent among the cattle. In the seventh book of Ovid's Metamorphosis there is an account of an epidemic that occurred sixty years before the Trojan war, in the island of Egina, in which the disease is represented as commencing first among birds, dogs, sheep, oxen, and then attacking mankind. Homer tells us that an epidemic that prevailed in the Grecian camp first commenced upon mules and dogs, and then attacked the human race. In Plutarch's Life of Romulus, we read of a pestilence which attacked cattle, and even affected vegetable life. Livy tells us that Rome was invaded three hundred years from its foundation by a terrible pestilence, in which cattle, as well as the human race, were victims.* Seventeen years later a similar mortality occurred among the

* Livy, lib. iii. 32.

cattle during the prevalence of an epidemic. Again, there was a similar occurrence in the year 353 from the foundation of Rome; also in the year 576.* Copland, in his *Medical Dictionary*, article Epidemics, considers this disease among cattle, noticed by Livy, as "having originated in malaria, and possessed the characters distinguishing this class of fevers." If this was the case, it is singular that along our Western rivers, where malarious diseases are so prevalent, and where they present the most malignant form, that cattle at the present day are not subject to such diseases; I am not aware that they are. The malady known as milk sickness frequently occurs on the high table-lands, where malarious diseases seldom if ever appear. Noah Webster, in his *History of Diseases*, has collected much information, not only in relation to epidemics, but also of diseases occurring among inferior animals. He tells us that "in the year 376 universal pestilence prevailed among men and cattle." He also tells us that in the year 810 "happened the greatest mortality among horned cattle that is on record. In some places in Germany it destroyed almost all the species." In the same work we are told that during the plague of 1348 "the death of animals, particularly sheep, marked this period. In England 5000 died in one pasture. The state of the air and water was so pestilential, that it is averred by historians that fowls and fishes had blotches on them." Hecker tells us, in his account of the "black death," "that as it advanced, not only men, but animals fell sick, and shortly expired, if they had touched things belonging to the diseased or dead." "Multitudes of dogs, cats, fowls, and other animals, fell victims to the contagion, and it is to be presumed that other epizootics among animals likewise took place, although the ignorant writers of the fourteenth century are silent on this point." Speaking of a fatal murrain that appeared among the cattle, he says: "Of what nature this murrain may have been, can no more be determined than whether it originated from communication with the plague patients or other causes." Copland, in *Medical Dictionary*, page 884, vol. i., states, upon the authority of Dr. Hodges, "that the plague of London, in 1665, was preceded by sickness among cattle." In 1669 a disease appeared among the cats at Westphalia. They died, Webster tells us, with an eruption on the head, accompanied with drowsiness.† He also tells us that "a mortal disease spread among cattle in 1682, in Italy, Switzerland, and Germany, that was called angina maligna, and of which cattle died in twenty-four hours. Authors relate that a blue mist appeared on the herbage of pastures. The disease moved about two German miles in twenty-four hours, and spread over Germany and Poland. Cattle at rack and manger were affected equally with those that grazed."‡ We are

---

* Livy, lib. iv. 21, 25; lib. v. 13; lib. xli. 21.

† Hist. of Diseases, vol. i. p. 204.

‡ Ibid., p. 204.

also informed in the same work, under the date of 1693, that "an epidemic catarrh began in Europe in October, being preceded by a similar disease among horses." Also in 1699, "a severe and fatal catarrh was epidemic, which carried off the young and robust, together with hard drinkers." At the same period, he tells us, "A cough was epidemic among horses in England and France." The same author tells us that in 1711, "a fatal distemper among cattle broke out, and raged with such violence in Italy as almost to destroy the species. It spread for three or four years, and horses perished by a similar pestilence. The writers who describe the disease represent it as a kind of plague." We are told in the same work, that in 1735 "a pestilential disease appeared in Devonshire, which swept away cattle and swine." We are told also, that between 1759 and 1763 was an unusually pestilential period, that during "this period fatal diseases carried off the cattle on the continent of Europe, and Toulon lost one-third of its inhabitants by an epidemic." "That in Europe the year 1763 was remarkable for diseases among various species of animals. In Denmark, an epidemic catarrhal disorder affected horses. In Madrid, a pestilence among dogs swept away multitudes: nine hundred died in one day. In Genoa, the poultry perished in a similar manner. In Italy, horses and swine fell victims to the pestilential principle. In France, horses and mules. In Sweden, sheep, horses, and cattle perished under the influence of the general cause." Again, "in 1785 canine madness spread in all parts of the Northern States; also, in 1797 a disease appeared among the cats, which swept away these animals by thousands." Copland says that during the epidemic in 1819, at New Orleans, "cattle, sheep, and horses were affected." (*Med. Dict.*, p. 884.) In the London *Lancet* for January, 1845, is a notice of the epizootic in Germany:—"This very disastrous disease originated in the plains of Russia, where it reigns epidemically. It is considered by the German veterinary surgeons to be a kind of contagious typhus. The various German States organized a strict quarantine on their frontier, but without success, for it has invaded Prussia and Austria, where it is said to be making great ravages." "In the eighteenth century several very fatal epizootics ravaged Europe from one end to the other. In 1754, such immense numbers of cattle were swept off, that several governments were obliged to forbid the destruction of any cattle capable of propagating the species." In the twenty-second number of the *British and Foreign Medico-Chirurgical Review*, is an interesting paper on the "Communicability of Cholera to Animals," by John Marshall, F.R.C.S. The writer presents many facts of much interest to the subject we have under investigation, as he enumerates the epizootics which have been supposed to be connected with cholera. He says: "In briefly reviewing our accumulated knowledge of reputed natural effects of cholera on the higher animals, we would in the first place dismiss, with the most passing notice, the numerous conjectures which are to be found in professional as well as non-professional records, as to the relation between the cholera and recent great and widely spread epizootics which have occurred in almost every country either before, concurrently with, or after the invasion of, the human pestilence. The widest and best known of these have been either influenza, like catarrhal, dysenteric, or pustular in their character, and identical with visitations previously well understood. The pleuro-pneumonia which has raged on the continent, and since 1838 in this country as so fatal an epizootic, and the typhoid disease of modern date, cannot, by any but the most rude analogy, be associated with human cholera."

"In the next place we would advert at somewhat greater length, but with equal reserve, to certain more partial or distinctly localized epizootics which have been noted in districts or towns of India and Europe, contemporaneously with the human pestilence. Thus in the Marquis of Hastings's army, in 1818, on the banks of the Sutlege, great numbers of oxen are said to have died suddenly during the prevalence of cholera, and from unknown causes. In the hilly districts, according to Chalmers, cholera being then very fatal in the towns, cattle died even in greater ratio than the people. In Rajpootana, according to Rankin, camels, and especially goats, died of diarrhœas and other ailments; and, on the authority of Mr. Searle, the same thing occurred in the Ladura and Cormbalosu districts. The disease among the poultry in Mr. Searle's compound may also be noticed here; and we are informed by an intelligent officer in the Indian army, that in 1832, at Rajahmundry, on the Godavery, all his ducks and geese died with symptoms which he and his native servants supposed to be truly choleraic. In Calcutta, in 1827, numbers of dogs died in the streets with choleraic symptoms; at Charcolly, also, fifteen-sixteenths of the dogs perished in the same way, during a second visitation of the disease, and at a later period half the dogs in Madras died with vehement vomiting and purging. At Macassar, in Amboyna, about the year 1818, monkeys, dogs, and oxen perished under similar circumstances; while quite recently (1849) horses died, as it was said, of cholera, at Penang, in great numbers. Merely glancing at the piscine mortality during the cholera period at Marienberg, which seems in one case to have been connected with the opening of a sewer into a pond; and alluding transiently, also, to the deaths of fishes in the rivers of Germany, and in the carp-ponds of the Departments of the Seine and Oise, in France, we find that at Astrachan, Moscow, and Warsaw, if not at Taganrog, in 1831, many examples occurred of poultry and other animals dying with symptoms resembling cholera. So, too, during the epidemic in Bohemia, according to Nekola, dogs died in a few days with diarrhœa and convulsions, preceded by loss of appetite and activity. In many parts of Austria the same circumstance was noted, and in Dantzic the cholera was ushered in by a canine epizootic of a choleraic form, so that dogs were said to be the first to indicate the approach of the disease. The Austrian Reports particularly mention poultry, dogs, and cats; and the French Report, by the Royal Academy of Medicine, on the Russian and Polish Epidemics, refers especially to the mortality among dogs. In 1832, cholera then raging in Paris, a sudden and singular mortality occurred among the poultry of the village of Choisi-le-Rois, situated on the banks of the Seine, about five miles lower down than Paris, though no cholera prevailed in the village at the time. The combs of the fowls attacked were cold and livid; a thready mucus appeared in the mouth and gullet; whitish liquid dejections were passed; the intestines were found red in patches, and the blood was thick and tarry. A similar epizootic was observed in two or three other villages in the Department of the Seine, and also on the Rhone. Since that period this disease of poultry has from time to time reappeared, but more particularly again in 1849–50, both in France and near Utrecht, in Holland, during the last cholera epidemic. Investigations and discussions as to its nature have been held by MM. Renault and Delafond Alfort, and the former concludes that the disease resembles cholera more than it does any other epidemic. It appears to be communicable by inoculation, and to affect rabbits also. In the winter and spring of 1831–2 a remarkable cholera-like epizootic, recorded by Professor Dick, occurred among cattle and horses in the neighborhood of Edinburgh, during the prevalence of cholera there, although it had somewhat preceded that disease in its earliest appearance. It is worthy of note that the same epizootic having apparently ceased during the cessation of cholera, reappeared near Leith, at the return of cholera in that place. Swine and dogs did not seem to be affected. The reputed horse-cholera, at Denny, in Scotland, in 1832, may also be alluded to in this place. In 1832–3, cholera then prevailing, a porcine dysenteric epizootic is on record in Ireland. In this category we would lastly include the unusual mortality, with more or less striking choleraic symptoms, among cats, goats, and dogs, mentioned in the journals of the times, as having been noted in Malaga, in 1834, and also in Algiers, Tunis, Cairo, and Constantinople, during the last epidemic visitation of the disease.

"Now, in reference to the majority of the examples which we have thus collected together of local epizootic diseases, more or less coincident with, and resembling cholera, it unfortunately happens that neither *the symptoms nor the post-mortem appearances have been made the subject of accurate medical investigation.* Hence we at once reject them as positive evidences of the effects of a cholera agent upon the animals concerned. And in regard even to those instances in which we have had the advantage of more precise medical observation, both before and after death, as in the case of the poultry by Mr. Searle and MM. Carrere Renault and Delafond, and in that of the epizootic described by Professor Dick,—instances which have been by some regarded as examples of cholera in animals,—we more than hesitate, on a careful consideration of the symptoms and morbid anatomy, to admit their sufficient analogy with the human disease." This summary of epizootics supposed to resemble cholera, increases the interest (when we see how little attention has been given to the subject) in the investigation of the present wide-spread disease among the swine ; a malady which has not only revived the name of cholera, but is generally believed by the public to be a species of this disease. The present period appears to be remarkable for the extensive prevalence of diseases among the inferior animals, for while the supposed cholera epizootic is prevailing among the hogs in this country, a fatal murrain is spreading among the cattle in Europe. An English paper says : "This terrible disease which is described by medical authorities as a pulmonary affection—a kind of induration of the lungs—differing in many respects from pleuro-pneumonia, highly contagious, and extremely fatal, has devastated part of Germany, and several smaller States bordering on the Baltic. Although it, or a similar deadly epidemic, scourged some of the midland counties in the last century, destroying entire herds with a rapidity resembling an aggravated form of cholera or plague in a populous city, yet, as a precaution against its introduction into the United Kingdom, an Order in Council was promulgated on Saturday last, prohibiting from that date the importation of cattle, horses, hoofs, and raw hides of cattle from certain infected districts on the continent. The importance of this timely and salutary measure, which has already been adopted by France, Prussia, and other European States, cannot for a moment be doubted, and we trust that, from our insular position, the distemper may be averted, and we be spared an impending calamity."

These brief notices of epizootics are sufficient to show that diseases among inferior animals, resembling epidemics in the extent to which they prevail, have occurred in all ages. They show, also, that it is highly probable that epidemic influences extend over *all* living bodies, and that there is an almost unexplored field for future investigation to ascertain whether the causes which spread an epidemic fever, the cholera, or influenza, over the globe, produce in the inferior animals similar diseases, or whether the influences which produce epizootics are the same as those which spread pestilence among the human race. It appears remarkable that in this age of research these subjects should have received so little attention. Wood, in his *Practice of Medicine*, article Epidemics, says: "The lower animals appear to be affected by epidemic influences, and seasons of unusual fatality among them have coincided with those in which the human race have suffered; but how far the effects are the same as upon man has not yet been accurately determined." Facts and observations on this subject would certainly assist in extending our knowledge of the nature of the epidemic influence. It requires no great degree of foresight to see that the time will come when epizootics, as well as every other phenomenon that is supposed to be either intimately or remotely connected with epidemics, will receive careful and thorough investigations. Subjects connected with human health will always receive the attention of mankind, and physicians will yet consider that it is not derogatory to their profession to enter the field, the stall, or the sty, and by the most careful and minute investigations endeavor to elicit from diseases of inferior animals facts that may throw light upon "the pestilence that walketh in darkness" and spreads desolation over the globe. The time will come, we repeat, when a remarkable epizootic will not be allowed to pass by, as we have witnessed within the last year, without investigation; not investigated alone by the veterinary surgeon, but by physicians, who will not only describe and record it as an event of the time, but trace its resemblance or relations to epidemics. With the full belief that such subjects are worthy the attention of physicians, I have collected a few facts in relation to our remarkable porcine epizootic, and endeavored to ascertain whether this disease was really a species of cholera, as it is supposed to be by the public, or whether it resembles any of the diseases to which the human system is subject. Should there be no notice taken of this malady among the swine—a disease which has borne the name of one of the most formidable diseases to which the human system is subject, and which has destroyed these animals by thousands and tens of thousands—it might be referred to at some future period as an evidence that cholera occasionally prevails among the inferior animals, or, at least, become as much a subject of conjecture as the epizootics that prevailed during the time of the "black death," or during some of the pestilential periods at Rome.

Authors have insinuated that epizootics have often "been exaggerated for the purposes of argument."* That some of the epizootics which occurred during the prevalence of a pestilence were exaggerated, is probable. When Plutarch tells us in his life of Romulus that, during the pestilence, "it rained blood in the city, so that their sufferings were increased with the terrors of superstition," we have our doubts with regard to the barrenness of cattle which he also represents as occurring. Gibbon, in alluding to this tendency to exaggeration, in his account of

* See Copland's Med. Dict., page 883.

the plague which prevailed between the years 250 and 265, says that "our habits of thinking so fondly connect the order of the universe with the fate of man, that this gloomy period of history has been decorated with inundations, earthquakes, uncommon meteors, preternatural darkness, and a crowd of prodigies fictitious or exaggerated." We know that what might be looked upon as vast numbers of animals dying during the prevalence of an epidemic, might be considered at another time as scarcely worthy of notice, and that there is much uncertainty and doubt with regard to the extent to which diseases have prevailed among inferior animals. To obtain information on this subject from our recent epizootic among the swine, and prevent it from being charged as exaggerated, I have collected some facts of the extent of country over which this disease has prevailed. The information obtained, though limited and probably falling far short of the real extent of this malady, is yet sufficient to show that it is wide-spread, and must, like an epidemic, depend upon some general predisposing cause.

I have seen notices of this disease prevailing in the States of Illinois, Kentucky, Indiana, Ohio, New York, Massachusetts, Pennsylvania, and Maryland. It has prevailed extremely in Indiana, particularly in Dearborn, Ohio, Ripley, Rush, Decatur, Brown, Bartholomew, Shelby, Johnson, Morgan, Marion, Boon, Posey, and Sullivan counties. It has also prevailed in Campbell, Kenton, Boon, Gallatin, Carrol, Breckenridge, Bullitt, Bath, Henry, Hendrix, Nicholas, Livingston, Union, and Crittenden counties, Kentucky. It has also prevailed in Hamilton, Butler, Clinton, Fayette, and Clermont counties, Ohio. Also, in different portions of Illinois, and very severely in Wayne, White, and Gallatin counties. It has also prevailed in the State of New York. The *Ohio Farmer*, for January 3d, 1857, quoting from the *Buffalo Republic* the extensive prevalence of the disease, says that, "in Western New York especially, we learn it has been very fatal, but is now over. In conversation with one of the most extensive dealers in the neighborhood, a day or two since, he informs us that about six weeks ago he lost about four hundred in a very short space of time. A distiller in Jordan, during the month ef September, lost fourteen hundred, which cost, in addition, over one thousand dollars to have them buried. In Rochester, at all the principal points, and even among the farmers, the mortality *has exceeded anything ever before heard of.* A butcher in this city not long since purchased five hundred dollars' worth of fat hogs, but they died so rapidly on his hands that he scarcely realized seventy-five dollars on the investment." "The *Worcester* (Mass.) *Spy* reports that many farmers in that city and vicinity are losing their swine by the mysterious and fatal disease known as the hog-cholera. In the southeast part of the town it prevails in a greater or less extent upon nearly every farm. In most cases the disease is traced to Western hogs that have been sold by the drivers the present season, and which seem to have communicated the contagion to the other inmates of the sties in which they have been kept. It is known that of many droves of Western shotes that have been sold at Brighton this season, and peddled about the State, nearly all have died." The disease has, no doubt, prevailed extensively in other parts of the country, of which I have seen no notice. In this section of the country it has been extremely fatal. Over portions of Dearborn County it spread from farm to farm, and some of our farmers lost from seventy to eighty out of the hundred of their hogs. At the distilleries the mortality has been very severe. I received information that more than eleven thousand died at the distillery in New Richmond, in the summer and fall of 1856. The owners of the distillery at Aurora inform me that they have lost between six and seven thousand. A gentleman informs me that he lost, in 1856, at Ingraham's distillery, in Cincinnati, from the first of August up to the twenty-fourth of October, one thousand two hundred and eighty-five,—losing 1152 out of a lot of 2408. Another gentleman informs me that at the distillery in Petersburg, Kentucky, he lost, from the first of June up to the eighteenth of October, 1856, 2576. I have also received information from several other distilleries where the losses were large. It was not, however, more fatal in the distilleries, in proportion to the number attached, than it was on the farms. The number of hogs that have died of this disease it is impossible to ascertain accurately. The *Cincinnati Gazette*, in giving the number packed at Cincinnati, for 1856–7, says, The crop was a short one by thirty-four per cent., as compared with the previous year, as shown by our returns, the accuracy of which has been again fully demonstrated. The whole number of hogs packed at this place last season and the former one compare as follows :—

| | |
|---|---|
| 1855–6 . . . . . . . | 405,393 |
| 1856–7 . . . . . . . | 344,512 |
| Deficiency for 1857 . . . . . . | 60,881 |

The *Cincinnati Price Current*, for February 17th, 1858, states that "the whole number packed at all the places reported last year, was 1,818,468, and the previous year 2,489,502," showing a deficiency in the West for 1857, of 671,034. This deficiency may to some extent be owing to the partial failure of the corn crop ; but we must bear in mind that the unusually high price of pork, and the fear farmers had of losing their hogs caused them very generally to dispose of all they could possibly make marketable. What number have been killed in the West during the last winter I have been unable to ascertain. I think, however, it will show a falling off in the ratio of increase which has taken place from year to year, corresponding with the increase of population and improvement of the country.

When the disease made its appearance in this section of country, in the summer of 1850, and we saw it spreading from farm to farm, the question was suggested whether it did not spread by contagion, for it was not known at that time (July) that the malady was contagious. Feeling much interest in watching the progress of this disease, from the large numbers of animals that were dying, I suggested to the owners of the distillery at Aurora, Messrs. Graffs, that we should try a series of experiments to ascertain the nature of the disease, and whether it was propagated by contagion. To this they readily assented, and as they were constantly receiving fresh hogs, there was a fine opportunity to make any experiments we saw proper. I am indebted to Mr. J. J. Barkman, of Aurora, for seeing that the following experiments, with the exception of the last, were carefully made:—The hogs on which the experiments were made, were known to be healthy.

1. Six hogs that had been exposed to the malady, by being in contact with diseased hogs, were put into a yard by themselves, and fed on slop and corn; on the fourteenth day from the time they were exposed to the disease, they were all unwell; three died within a week afterwards; the rest recovered.

2. Ninety hogs were exposed to the disease, then put into a yard by themselves, and fed on corn and water, (no slop given;) in thirteen days disease made its appearance among them, and they continued to die until sixty out of the ninety died.

3. Fifty hogs were put into a pen by themselves, and fed on slop; they had not been exposed to the disease; for six weeks they continued healthy.

4. One hundred hogs that had not been exposed to the disease were put into a yard by themselves, and fed on corn and water; for thirty days no symptoms of disease appeared among them. They were then put into a pen with diseased hogs; on the thirteenth day they began to show symptoms of the malady, and the disease rapidly spread among them, until forty died.

5. Thirty-three hogs, out of a lot of two hundred and sixty-three, were put into a pen by themselves; for six weeks they continued healthy. The remaining two hundred and thirty were put into a pen adjoining which were diseased hogs; in thirteen days disease made its appearance among them, and continued until one-half died.

6. Four young and healthy hogs were put into a pen in which, four days previous, diseased hogs had been; they were fed on corn and water. On the fourteenth day they were all unwell, and one died on the fifteenth day, and in five days more they were all dead. This experiment shows that the infection may be retained in a pen several days.

7. I inoculated, on the 28th of October, five healthy hogs with the blood taken from the inflamed tissues of hogs that had died of this disease. On the fourteenth day (November 11th) they were all unwell, and all died with the exception of one. In three inflammation spread from the point where they were inoculated, along the skin and down the legs, which became very much swollen. I cannot say that this inflammation was caused by the inoculation, for it did not appear until the fourteenth day, and many hogs had this external form of disease.

From these experiments I think that we not only ascertained that this disease was infectious, but that the infection had a latent period of from twelve to fifteen days. Observations have since led me to consider the latent period as varying from twelve to twenty days. These experiments also showed that the hogs in the pens were not dying from strychnine in the slop. A statement was going the rounds of the papers about this time, that strychnine was used in making yeast at the distilleries, and was poisoning the hogs at these places in large numbers.

The manner in which this disease in many instances spread among the hogs from farm to farm, also showed most conclusively that it was infectious. One farmer had seventy-five hogs that he turned into a corn-field to fatten. These hogs had been exposed to the disease, had become sickly, and numbers had died. He bought thirty-six more; these were all healthy; they were put into the same field with the diseased hogs; in two weeks they were unwell, and numbers died. He bought forty-five more, all healthy, and put them in the same field. In two weeks they began to show symptoms of disease, and in a few days after a number died. Finding that he was likely to lose all his hogs, he sold fifty of the fattest that were left to the butchers in Cincinnati, at a reduced price, as diseased hogs. These hogs, it was said, were purchased for their fat, to be used in the manufacture of lard oil. After deducting the fifty he sold, he lost out of the one hundred and fifty-six, all but ten. A large number of facts could be given, if necessary, to show the contagiousness of the disease. Hogs that have been once under the influence of this malady, appear not to be susceptible to a second attack. Although placed in pens with diseased hogs, they continue healthy and fatten, and not a single instance can I find of hogs having this disease twice. There is reason to believe that this malady occasionally prevails at the distilleries in a mild form, as the old hogs at many of these places did not take the disease. But why it should suddenly have assumed so malignant a character, is as difficult to account for as that scarlatina, from being at one time the mildest of diseases, at another becomes one of the most malignant. On the farms the old and young hogs appear to be equally susceptible to the disease.

I endeavored to ascertain where this malady originated. I found, on inquiry, that on June 19th, 1856, one hundred and three hogs were brought to the "pens" at Aurora, from Ripley County; these hogs were unwell at the time, and some of them died the next day after they were brought in. The hogs in the pens up to this time were healthy. After these diseased hogs had been put into the pens, they continued to die from one to five a day, until about one-half died. In two weeks the disease appeared in the adjoining pens, and by the latter part of July the hogs were dying from ten to twenty a day. Nearly every hog in these pens—about four thousand—took the disease, and thirty-three per cent. died. After the disease appeared in a pen, (each pen contains a hundred hogs,) it generally was from six to eight weeks before the hogs that were left recovered. A gentleman who resides in Ripley County, on the second farm from where these hogs were brought, informs me that he lost eighty out of one hundred and fifty, and that his neighbor, who lives on the adjoining farm, lost about thirty, and that the disease was prevailing in this neighborhood before the hogs were taken to Aurora. On these farms, then, in this section of country, I think we may consider as one of the places in which the disease originated. I cannot ascertain how it was introduced into this neighborhood. These farms are in the interior of Ripley County, away from the river; not near the railroad, or any very public thoroughfare; nor within twenty miles of a distillery. I think that we have evidence, also, that it originated in several places in Boon County, Kentucky; that it also occasionally originates within the pens at distilleries, I think is highly probable. There were four thousand old hogs in the pens at Aurora while the malady was prevailing that continued healthy, which I cannot account for, except by considering that they had been under the influence of this disease at some previous period. The introduction of the disease into the pens at the distillery in Rising Sun, in Ohio County, was traced to hogs purchased of a farmer in Kentucky. This farmer was sued for damages. A gentleman who was fattening hogs at the distillery at Patriot, in Switzerland County, informs me that when he saw the hogs were dying in the country, he refused to buy more, and his hogs continued healthy. I mention these facts, as it has been stated in the public papers that this disease originates within the distilleries. I was inclined to believe so at first myself; I thought it was produced from the slop, and by crowding large numbers of hogs together. But I have become fully satisfied that although the disease may occasionally arise within the pens at distilleries, it also originates among hogs running at large on farms. When we see the possibility of large quantities of diseased meat finding its way into market—meat, too, of animals laboring under a most infectious malady—which may produce injurious effects upon human health, the causes which produce this disease become worthy of very careful investigation. And also, if by actual experiment we can ascertain what gives rise to infectious diseases among inferior animals, it may by analogy throw some light upon the manner in which this class of human diseases originates.

This disease, then, appears, like some of the human pestilences, to occasionally originate spontaneously, or from unknown causes, but when once produced spreads rapidly by infection. It was too widely extended to be spread over the country (on opposite sides of the Ohio River) by contagion alone. Although it appeared to prevail in certain localities or neighborhoods like cholera, still it differed from this disease in the infection having a more definite or uniform period before it became manifest.

On examining the books that treated of diseases of domestic animals, I found swine were represented as being subject to fever, leprosy, murrain, measles, jaundice, foul skin, mange, staggers, chacklins, swelling of the spleen, surfeit, lethargy, heavings, diarrhœa, quinsy, tumors, catarrh, and also tubercles. The descriptions of these diseases were extremely brief and unsatisfactory, and none of them represented the symptoms of this epizootic. At first I thought it was a malignant form of measles; but the symtoms did not resemble those described as belonging to this disease. From the diarrhœa, and the rapid manner in which in many instances it terminated fatally, the public gave it the name of cholera.

This disease presents a great variety of symptoms. In January, 1856, when this epizootic was at its height in this section of country, I published a short notice of it in the *Cincinnati Gazette*. The symptoms which I then described I have found upon a more extensive observation to be correct. The hog at first appears weak, his head droops, and sometimes in a few hours after these symptoms, diarrhœa commences. There is frequently vomiting. In some cases the discharges were serous and clay-colored, sometimes dark, also bloody, and mucous resembling those of dysentery. The urine at first was generally small in quantity and high colored, but as the animal recovered it became abundant and clear; this was one of the symptoms by which the men, who were attending the hogs at the distillery, ascertained that they were recovering. *In a large number of cases the respiratory organs appeared to be principally affected;* there was coughing, wheezing, and difficult respiration. In some instances the animal lost the power of squealing, and the larynx was diseased. There was frequently swelling of the tongue, and bleeding from the nose. In those cases where the respiratory organs were the principal seat of the disease, there was generally no diarrhœa or dysentery. In many instances the disease appeared to be principally confined to the skin; sometimes the nose, the ear, or the side of the head, were very much inflamed; the ear swollen to twice its usual thickness. This inflammation would spread along the skin sometimes over the eye, producing complete blindness. Sometimes one or more legs were inflamed and swollen, and the inflammation also extended along the body. The skin where it was inflamed was red and swollen. Some had large sores on their flanks or sides from three to six inches in diameter. In one instance, at the distillery, the inflammation extended along the fore leg, the foot became ulcerated and sloughed off, and the animal recovered. Some appeared delirious, as if there was inflammation of the brain. I examined the blood of four hogs which had this disease well marked; they were stuck, and the blood, arterial and venous, was caught in a bowl. It was cupped and presented a well-marked buffy coat. Death took place in from one to ten days after the attack. Sudden changes in the weather, particularly from warm to cold, appeared to increase the fatality of this disease. The average mortality of hogs that were in pastures or fed on slop, was from thirty-three to forty-five per cent., but it was frequently much more fatal if hogs were fed on corn,—in some instances ranging from seventy to eighty out of the hundred, and in some instances even higher.

I found, on opening the bodies of hogs that had died of this disease, that they all presented evidences of a diffusive form of inflammation. From sixty-seven hogs that I have examined, I found it was not confined to any particular tissue. Sometimes this inflammation was confined to one organ, in other cases it attacked several at the same time. The skin frequently presented patches of inflammation, and often had a purple appearance. In cutting through parts that were the most inflamed, the skin was swollen, and the cellular tissue was infiltrated with serum. Frequently, however, the skin was merely discolored, without any swelling whatever. The stomach was occasionally distended with food, and the mucous membrane in nearly every instance presented evidence of inflammation, sometimes extending over the whole stomach, at others only in patches; it was generally of a deep red color, thickened, and frequently softened. Sometimes it was covered with a viscid mucous, in other instances there was an effusion of blood into the stomach. The mucous membrane of the small or large intestines, where there had been diarrhœa or dysentery, presented in all instances evidences of inflammation; in patches it was red, thickened, sometimes softened, and occasionally ulcerated; where there had been dysentery there was generally bloody mucus found in the large intestines. The bladder generally contained urine; sometimes its mucous membrane was inflamed, and in one instance there was an effusion of blood into this organ. In a large number of cases I found evidences of peritoneal inflammation, such as redness of this membrane, effusion of turbid or bloody serum, adhesions between the intestines, and between the intestines and sides of the body. In three instances blood was effused into the peritoneal cavity—in one instance more than a quart; it appeared in this case to come from the liver. The liver was occasionally the seat of this inflammation, not only in its inverting membrane, but the parenchyma; sometimes there were abscesses, and in one instance portions of it were gangrenous. The lymphatic glands were generally of a dark red color, frequently resembling clots of blood. This disease of the lymphatic glands was of common occurrence.

The lungs were frequently the seat of this inflammation, portions of one or both presenting different appearances from simple congestion to complete hepatization; sometimes there was ulceration, and frequently there was a turbid or sero-purulent, or bloody effusion into the pleural cavity; sometimes there were extensive adhesions between the lungs and pleura of the ribs. At first I was inclined to believe this malady to be a form of pleuro-pneumonia, bút after I became better acquainted with it, I found that the inflammation was not uniformly confined to any organ. In a number of instances the mucous membrane of the bronchia was deeply in-inflamed, and the inflammation extended to the trachea and larynx. In several instances the larynx was inflamed, resembling laryngitis. One animal that had great difficulty in breathing, and could make no noise, I had knocked in the head, and on examination, I found the mucous membrane of the larynx and epiglottis inflamed and swollen; also the tongue was swollen. There were evidences in several instances of pericarditis, which had produced adhesions between the heart and pericardium. The brain, from the difficulty of opening the skull, was examined only in one instance; it was found healthy, although I feel confident it was frequently the seat of the disease.

From these examinations we see that it is a misnomer to call this malady cholera. It is a contagious inflammatory disease, the inflammation being confined to no particular tissue, sometimes attacking only one, at others, several in the same animal. Evidences of this inflammation were found in the dermoid, the cellular, the serous, the mucous, and glandular tissues. I consider it a diffusive form of inflammation from the manner in which I have witnessed it spread along the skin. In one night I have seen it extend from the eye to the ear—the ear becoming inflamed and swollen. Although we have not been able to show that this is a cholera epizootic, still the facts elicited may be of interest, and remove doubts at some future period. But, then, if this malady does not resemble cholera, does it resemble any of the diseases to which the human system is subject? I think not. Like the specific eruptive diseases, it is highly contagious; the infection has a period of incubation of from twelve to twenty days, and one attack appears to exempt the animal from a second. But in this disease, although petechia and an eruption may appear on the skin, its principal characteristic is a diffusive form of inflammation, which may attack nearly every tissue and spread like an erysipelas. But then, again, it differs from this disease, as it is well known that in erysipelas one attack does not exempt the system from a second; and although erysipelas may be contagious, still it is doubtful whether the period before the eruption shows itself is so uniform as in this disease; and while erysipelas is generally confined to the skin, this inflammation most frequently attacks the lungs and mucous membrane of the alimentary canal. This disease appears to be intermediate between the specific eruptive diseases and erysipelas—partaking of the nature of each, and probably not having its exact resemblance among the diseases to which the human system is subject.

This epizootic has probably been the most extensive that ever appeared in our country. The question is continually suggested what causes have predisposed the swine to this disease. Has it spread from that epidemic influence which has recently favored the extension of cholera over the globe? or have the remarkable seasons which we have had within the last three years, given rise to, and favored the spread of this malady? Since 1855 we have had unusual fluctuations in the temperature, and humidity of the atmosphere; such fluctuations as some authors would consider as belonging to pestilential periods. We understand how, in the *regular* order of nature, the revolution of the earth, and unequal temperature at different latitudes produced by the sun, give rise to great aërial currents; the prevailing winds, and the great storms which deposit moisture, absorbed by the atmosphere from the Southern Ocean and Gulf, over large portions of our continent. But I am not aware that any successful attempt has yet been made to account for these extreme fluctuations in temperature and humidity; fluctuations which have been observed in all ages and nearly all countries, producing in ancient times the famine, and supposed to be intimately connected with the causes which spread pestilence over the world. A brief notice of these extremes in seasons in this country, as a possible cause of this epizootic, and their effects upon human health, may not be out of place. The winter of 1855–6, as is well known, was intensely cold. The Ohio River was frozen over, and during the month of January loaded wagons were crossing and recrossing daily on the ice. This cold winter was followed by an unusual drouth, which commenced early in the spring, and continued through the summer; some of the crops were almost a complete failure; thousands of acres of grass and small grain in this section of country were not even harvested; the springs throughout the country became dry; the river receded to its lowest mark; navigation was almost entirely suspended, and the river closed again in the winter of 1856–7, which was also remarkably cold. The following spring was at least a month later in this latitude than usual, and frost and snow appeared in the Southern States as late as April. I have seen it stated that such an event had not been known before for fifty years. The last summer has been unusually propitious; the temperature has been mild, with an abundance of rain; vegetation has been luxuriant; the crops of all kinds have been unusually large; and, although the corn-rot has been extensive, there probably never was a greater abundance of agricultural products in the country than at present. The winter of 1857–8 has been as remarkably mild, up to February 10th, as the other two winters had been unusually severe. On the 20th of January the maples were out in blossom, many of the flowers were up, the robins and other spring birds were here in abundance, and the temperature more resembled April than January. During these remarkable seasons, the country, since the fall of 1855, has been unusually healthy. No fatal epidemic has prevailed; neither the cold winters, or the extreme drouth of 1856, or the wet and fruitful summer of 1857, or the present mild winter, has produced any extensive disease among the human race. Those who consider that "extremes of temperature, whether high or low, are eminently destructive to life," will find it difficult, I think, if we except the epizootic among the swine, to reconcile their conclusions with the remarkably healthy seasons which have recently occurred in connection with the extremes of temperature. Scarlatina and measles prevailed in the neighborhood of Aurora in the spring of 1857, and measles were more prevalent than they had been for many years. Still, *these epidemics were local*, and the cases so mild that few required medical treatment. The mild form which measles assumed may have caused, to some extent, their general prevalence; as parents were induced, from this mildness, in many instances to expose their children to the disease. Are we, then, to consider this epizootic as arising from these extremes of temperature, or humidity of the atmosphere? If so, why should it prevail with equal fatality in very opposite seasons—prevailing in the dry summer of 1856, also in the cold winter that followed, and also prevailing in the wet summer of 1857? Are we to consider it connected with the causes which produce epidemics? If so, why has the human race been so unusually free from disease? If there had been an epidemic influence during the last two years, would we not have seen more or less of its effects upon the community, and why were not the swine attacked when epidemics were prevailing in the country? Have not the conclusions been too hastily adopted that regard the epidemic influence as extending over all organized bodies, or even over the inferior animals? That the vital force extending through all living bodies is in a continual perturbation, arising from changes incessantly taking place in physical nature, there can be no doubt. The storms and calms, the low or elevated temperature of certain years, the dry or humid atmosphere, are constantly modifying the conditions of living bodies, but with widely different effects. An elevated temperature and humid atmosphere, which gives rise to deadly miasms, spreads disease among the human race, favors the luxuriant growth of the vegetable world, and gives increased power and activity to not only insect life, but to many of the inferior animals. The causes which favor disease in one species of animals seem to give fresh life and vigor to another; as the carrion on which one species would fatten, would be to another poison. Whether the epidemic influence extends over all living bodies, and depends upon some unknown magnetic or electrical condition of our globe, or whether it arises from meteorological changes, or causes beyond, which bring about these changes, will be subjects of conjecture until phenomena are more extensively and carefully observed. We have seen that this epizootic has prevailed extensively, and spread over the country similar to an epidemic; and would not the theories which have been advanced to account for the cause and spread of epidemics equally apply to the cause and extension of this disease, whether we regard it as fungi, animalculi, electrical, atmospheric, a deficiency of ozone, or produced from an influence of the heavenly bodies? Does not the spread of this epizootic have rather a tendency to show the fallacy of some of these theories, and does it not also show that it is highly probable that, as we become more thoroughly acquainted with these wide-spread diseases among inferior animals, we may find that they are confined only to species, and that they arise from causes as distinct from those which produce a human pestilence, (although they may occur simultaneously,) as the potato-rot does from those which produce the yellow fever?

Another question connected with this subject, and one of much interest, is in the possibility of diseased meat finding its way into market, and whether its consumption would produce injurious effects upon human health. The rapid manner in which this malady spreads through a drove of hogs, the large numbers that die, and the severe losses which have been sustained from this disease, are now strong inducements for persons inclined to act dishonestly, to either butcher or dispose of their hogs as soon as this disease makes its appearance among them. The fifty hogs already alluded to, that were disposed of by one of our farmers, is an instance. This farmer acted honestly, however; he sold them at a reduced price as diseased hogs; but if the butchers to whom he sold them had not been honest men, this meat would probably have been sold in market. The *Baltimore American* says the mayor, accompanied by a deputy marshal, visited a distillery, "and there saw a considerable number of men all busily engaged in cleaning hogs, and preparing them for the parties that had already bargained for the two furniture wagon loads which were captured on the previous night by the police of the southern district. They counted no less than forty-one hogs, many of which bore unmistakeable evidences of death either from violence or disease. The bodies were highly discolored, and the smell arising therefrom was of the most offensive nature. It was the intention of the authorities to have arrested all the parties, but they fled ere they could be taken. Yesterday an officer, acting with the authority of the Board of Health, confiscated all the hogs, and will take such measures hereafter as will prevent such traffic, at least in that quarter. There is a very general feeling in existence in relation to the whole affair, and memorials are now in circulation asking for the appointment by the councils of regular meat inspectors for the city markets. It is stated that upward of *six thousand diseased hogs have already been* DISPOSED *of in this city*, by certain parties. Such transactions will have a tendency to increase the business of the respectable victuallers, for persons will rarely, with any degree of security, purchase of any others." The *N. Y. Post* says: "The hog cholera is creating an excitement not only out West, but in the Eastern States. At Brighton, on Monday last, the alarm was so great, that 2500 hogs offered for sale, met with no buyers at any price. In Greenbush, we understand that the sick hogs arriving from Illinois and Ohio amount to one hundred and fifty a day. A large number of them are *slaughtered* in the village, and sent to New York and Boston." I have also seen notices in the public papers in this State, of persons supposed to have been made sick by eating this diseased meat. Independent of the disgust arising from the idea of eating flesh of diseased animals, what effect would this meat have on health? The following facts may probably be of some interest on this subject. A dog belonging to Mr. Wm. Rickets, of Aurora, was chained near the pens at the distillery, and fed on diseased meat; he continued healthy and grew fat, until the sixth week, when he became unwell, vomited frequently a greenish looking mucus, and died on the third day from the time he first showed symptoms of disease. Two more dogs, both belonging to Mr. Woulf Denderline, of Aurora, were also chained near the pens, and fed on diseased meat. One continued healthy until the fifth week, when he became unwell, refused to eat, vomited also a greenish fluid, had diarrhœa, and died on the sixth day from the time he was taken unwell. The other dog continued well until the fourth week, when he was attacked with vomiting; no diarrhœa was observed, and, on the third day after, he died. Two more dogs, belonging to Mr. John Buffington, were chained at the pens, and fed on diseased meat. One of them died in the third week, with similar symptoms to the first mentioned. The other became unwell on the fourth week. The owner thinking that he was going to die, had him removed, and fed on different food. He gradually recovered, though it was with difficulty that he could walk for more than a month afterwards. I saw him three weeks after he had been removed from the pens. On making him get up, he would stand with his head down, his body drawn up, his back arched, and in attempting to walk, would put one foot an inch or so beyond the other. One of the men who attends the hogs informs me that for sixteen years he had been engaged in this business, and always kept a dog chained near the pens, which was fed on the flesh of the hogs that died, and that this food never before injured his dogs. At first I was inclined to believe that the consumption of this meat would not be directly injurious, but from the effects which we have witnessed upon these dogs, it appears highly probable that, used as an article of food, this meat may gradually produce in the human system diseases the source of which would scarcely be suspected. When we consider the large amount of pork that is consumed in our country, this question becomes one of deep interest; and although the facts which I have presented may not be conclusive as to the poisonous character of this meat, still they show, at least, that this subject is worthy the attention of physicians, and a more careful investigation than it has received from me. I may mention here, that while making my examinations (although careful not to have any hogs opened except those that had died with well-marked symptoms of this epizootic) I frequently found chronic diseases which appeared to be of long standing. Sometimes there were an immense number of tubercles in the lungs, also occasionally in the liver. In some instances there were abscesses in the liver, also in the mesentery, filled with yellow pus. Sometimes there were worms in the intestines, also in the kidneys, and diseases sufficient to show that, besides the filthy habits of the swine, the Jew and the Turk have reasons for regarding the flesh of this animal as unclean.

From the very infectious character of this epizootic, the question may be asked, could this disease be communicated to the human system? Copland, in his *Medical Dictionary*, page 882, says: "Epidemics often commence among the lower animals, especially horned cattle and sheep, and the use of the diseased flesh may occasion malignant diseases among the human species. Whether or not infection may be conveyed from these animals, while alive, to man, during epizootic, has not been fully ascertained. That it can be thus conveyed in respect to some maladies, has been proved in modern times." We have no evidence that this disease among the swine can be communicated to the human system. The men who attend these animals at distilleries, have spent nearly their whole time, for months together, surrounded by hundreds of diseased hogs, and yet, among these men at all the distilleries I can hear from, there has been no unusual sickness. Also the soap-boilers and their assistants, who are constantly handling the diseased hogs, and breathing the effluvia arising from their putrefying bodies, often intolerably offensive, do not appear to have been affected. While examining the diseased hogs, I several times wounded myself; but the wounds readily healed, and there was no unusual inflammation. This disease seems to be peculiar to the swine, and it is extremely doubtful whether, under any circumstances, it can be communicated to the human system.

Those who feel an interest in this epizootic, may find a more full account of it in the forthcoming volume of the *Transactions of the Indiana State Agricultural Society*.

